# Supplementary material for: Modeling regulatory network topology improves genome-wide analyses of complex human traits
Source: Nat Commun. 2021 May 14;12:2851. doi: 10.1038/s41467-021-22588-0 (PMC8121952; doi:10.1038/s41467-021-22588-0)
Supplement: Supplementary file 2 — Reporting Summary [file 41467_2021_22588_MOESM2_ESM.pdf]

nature research

Corresponding author(s): Xiang Zhu

Last updated by author(s): Feb 20, 2021

Reporting Summary

Nature Research wishes to improve the reproducibility of the work that we publish. This form provides structure for consistency and transparency in reporting. For further information on Nature Research policies, see Authors & Referees and the Editorial Policy Checklist .

Statistics

For all statistical analyses, confirm that the following items are present in the figure legend, table legend, main text, or Methods section.

n/a	Confirmed	
☐	☒	The exact sample size (n) for each experimental group/condition, given as a discrete number and unit of measurement
☐	☒	A statement on whether measurements were taken from distinct samples or whether the same sample was measured repeatedly
☐	☒	The statistical test(s) used AND whether they are one- or two-sided<br>*Only common tests should be described solely by name; describe more complex techniques in the Methods section.*
☐	☒	A description of all covariates tested
☐	☒	A description of any assumptions or corrections, such as tests of normality and adjustment for multiple comparisons
☐	☒	A full description of the statistical parameters including central tendency (e.g. means) or other basic estimates (e.g. regression coefficient) AND variation (e.g. standard deviation) or associated estimates of uncertainty (e.g. confidence intervals)
☐	☒	For null hypothesis testing, the test statistic (e.g. F, t, r) with confidence intervals, effect sizes, degrees of freedom and P value noted<br>*Give P values as exact values whenever suitable.*
☐	☒	For Bayesian analysis, information on the choice of priors and Markov chain Monte Carlo settings
☐	☒	For hierarchical and complex designs, identification of the appropriate level for tests and full reporting of outcomes
☐	☒	Estimates of effect sizes (e.g. Cohen's d, Pearson's r), indicating how they were calculated

Our web collection on statistics for biologists contains articles on many of the points above.

Software and code

Policy information about availability of computer code

Data collection	No software was used in the data collection of this study.
Data analysis	The RSS-NET software is available at https://github.com/suwonglab/rss-net (DOI 10.5281/zenodo.4553387). Other software packages used are RSS-E (accessed October 19, 2018), Pascal (accessed October 5, 2017), LDSC (version 1.0.0, accessed November 27, 2018), PECA (accessed July 12, 2018), MACS2 (accessed July 12, 2018) and HOMER (accessed July 12, 2018). The links and identifiers of all codes and usage of all software packages are described in Methods.

For manuscripts utilizing custom algorithms or software that are central to the research but not yet described in published literature, software must be made available to editors/reviewers. We strongly encourage code deposition in a community repository (e.g. GitHub). See the Nature Research guidelines for submitting code & software for further information.

Data

Policy information about availability of data

All manuscripts must include a data availability statement. This statement should provide the following information, where applicable:

- Accession codes, unique identifiers, or web links for publicly available datasets
- A list of figures that have associated raw data
- A description of any restrictions on data availability

The 38 network files are available at https://github.com/suwonglab/rss-net (DOI 10.5281/zenodo.4553387). Analysis results of 38 networks and 18 traits are available at https://suwonglab.github.io/rss-net/results. Links and identifiers of other data are specified in Methods, Supplementary Notes 5 and 8. Source data are provided with this paper.

Field-specific reporting

Please select the one below that is the best fit for your research. If you are not sure, read the appropriate sections before making your selection.

- [x] Life sciences
- [ ] Behavioural & social sciences
- [ ] Ecological, evolutionary & environmental sciences

For a reference copy of the document with all sections, see nature.com/documents/nr-reporting-summary-flat.pdf

Life sciences study design

All studies must disclose on these points even when the disclosure is negative.

Sample size	Sample sizes of GWAS summary statistics used in this study were predetermined by the corresponding publications (Supplementary Note 8 and Supplementary Table 1).
Data exclusions	No data were excluded from the analyses.
Replication	Replication was not feasible because both GWAS summary statistics of the same traits and gene regulatory networks of the same contexts that were generated from independent samples were not available at the time of analysis.
Randomization	Randomization was not relevant as the study is observational.
Blinding	Blinding was not relevant as the study is observational.

Reporting for specific materials, systems and methods

We require information from authors about some types of materials, experimental systems and methods used in many studies. Here, indicate whether each material, system or method listed is relevant to your study. If you are not sure if a list item applies to your research, read the appropriate section before selecting a response.

Materials & experimental systems

n/a	Involved in the study	
x		Antibodies
x		Eukaryotic cell lines
x		Palaeontology
x		Animals and other organisms
x		Human research participants
x		Clinical data

Methods

n/a	Involved in the study	
x		ChIP-seq
x		Flow cytometry
x		MRI-based neuroimaging
