# Supplementary material for: Modeling regulatory network topology improves genome-wide analyses of complex human traits
Source: Nat Commun. 2021 May 14;12:2851. doi: 10.1038/s41467-021-22588-0 (PMC8121952; doi:10.1038/s41467-021-22588-0)
Supplement: Supplementary file 3 — Descriptions of Additional Supplementary Files [file 41467_2021_22588_MOESM3_ESM.docx]

Descriptions of Additional Supplementary Files

**Supplementary Data 1**

**Description:** Information of paired gene expression and chromatin accessibility data from 207 biosamples that are used to construct 38 TF-TG regulatory networks for this study.

**Supplementary Data 2**

**Description:** Correlations between 73 binary functional annotations in LDSC baselineLD v2.1 and log 10 enrichment BFs, either across 512 trait-network pairs that pass the near-gene control or across all 684 trait-network pairs (18 traits and 38 networks). For a given functional annotation, we estimate the correlation between log 10 BF and proportion of SNPs falling into both a network and this functional category, across all trait-network pairs. Rows are ranked by two-sided Pearson P-values based on 512 trait-network pairs. The Bonferroni cutoff is 0.05/73=6.8E-4.

**Supplementary Data 3**

**Description:** Correlations of log 10 enrichment BFs between 18 traits across 38 networks. Rows are ranked by two-sided Pearson P-values. The Bonferroni cutoff is 0.05/153=3.3E-4. Trait abbreviations are defined in Supplementary Table 1. Genetic correlations computed on the same GWAS summary data are retrieved from https://atlas.ctglab.nl/ (accessed June 8, 2020).

**Supplementary Data 4**

**Description:** Overlap between RSS-NET prioritized genes (ܲଵୠ୫ୟ ≥ 0.9) and genes implicated in 27 categories of knockout mouse phenotypes (http://www.informatics.jax.org/, accessed November 28, 2019) for 14 GWAS traits. The 14 traits are obtained by removing 2 disease subtypes (MI for CAD, UC for IBD) from the 16 traits that pass the near-gene control. Analysis details are provided in Supplementary Figure 15. Within a trait, rows are sorted by two-sided Fisher exact P-values and then odds ratios from the analysis of all genes (the 4th column). Trait abbreviations are defined in Supplementary Table 1. We use the 1st row "41 / 184 (19.1726)" to explain the meaning of "GWAS overlap" (the 3rd column): 184 genes are implicated by GWAS of AF in the GWAS Catalog at the time of analysis, 41 of them belong to the "Muscle" category, and the negative log 10 twosided Fisher exact P-value is 19.1726.

**Supplementary Data 5**

**Description:** Overlap between RSS-NET prioritized genes (ܲଵୠ୫ୟ ≥ 0.9) and genes causing 19 categories of Mendelian disorders (https://github.com/bogdanlab/gene_sets/, accessed November 28, 2019) for 14 GWAS traits. The rest is the same as Supplementary Data 4.
